# People’s Attitude and Perception of the Pandemic on Twitter: A Case Study of COVID-19 in India

**DOI:** 10.7759/cureus.76320

**Published:** 2024-12-24

**Authors:** Meera PV, Dhivya Karmegam, Suriya Saravanan

**Affiliations:** 1 School of Public Health, SRM Institute of Science and Technology, Kattankulathur, IND; 2 Department of Civil Engineering, Mepco Schlenk Engineering College, Sivakasi, IND

**Keywords:** covid-19 outbreak, emotional analysis, text mining, topic modelling, twitter

## Abstract

Background

Understanding the attitudes and perceptions of the general population is necessary for organizing health promotion initiatives. During outbreaks, social media has a significant impact on creating social perceptions. This study aims to identify and examine the emotions expressed and topics of discussion among Indian citizens related to COVID-19 third wave, from the messages posted on Twitter using text mining techniques.

Methods

Twitter messages (tweets) were downloaded using Twitter API from June 1, 2021 to July 10, 2021. After pre-processing the downloaded messages, 8933 unique tweets from various individuals were taken into account for the analysis. To identify and extract emotions expressed by the people in the Twitter texts, the text mining and sentiment analysis package “syuzhet” in R software was used. In order to identify the concerns and themes of discussion by the public during the pandemic, topic analysis was done using the Latent Dirichlet Allocation (LDA) technique. To understand and measure the tweets' reachability in relation to the themes and emotions conveyed, an engagement metrics analysis was also conducted.

Results

According to the emotional analysis performed on Twitter messages about the COVID-19 third wave, anticipation was exhibited maximum in 4180 tweets, followed by fear in 4070, and trust in 4001 tweets. Results of topic modeling revealed that there were widespread discussions and concerns about preventative measures to deal with the COVID-19 third wave. Engagement metrics verified that the greatest number of individuals liked and retweeted tweets expressing disgust. Maximum people favorited tweets with information on preventive measures for COVID-19, and a large number of individuals re-shared messages comparing various aspects of different COVID-19 waves.

Conclusion

Data from social media platforms can be used to comprehend public opinions and emotions during pandemics and emergencies. This can assist public health stakeholders in managing pandemic circumstances by planning effective health communication strategies that quickly reach a larger audience. The study's findings will assist stakeholders and public health experts in effectively using social media to communicate information about COVID-19 that combat people's negative emotions and concerns. Future research can use a similar approach to comprehend people's perspectives and concerns during outbreaks and emergency circumstances

## Introduction

COVID-19 is a dreadful disease that threatens populations all over the world. Across the globe, the COVID-19 pandemic has had a major influence on social, economic, and health conditions. Many places imposed lockdowns and other limitations to stop the spread, which had a severe impact on businesses and daily life. In India, after the first and second waves of COVID-19 infection, studies predicted that there was more possibility that India may be hit by a third wave of infection in July - August 2021 [1]. At that point in time, many countries across the globe have already experienced COVID-19 third wave [2,3]. Regardless of the third wave’s likelihood and potential severity in India, it is critical to be prepared to handle the pandemic. An early and appropriate intervention will help in the effective management and mitigation of pandemics. High vaccination coverage, social distancing, face mask usage, self-quarantine, personal hygiene, and restrictions on public gatherings are some of the strategies that were implemented to suppress the COVID-19 third wave in India [4].

Considering the density and range of the population in India, implementing physical distancing and educating citizens to use masks can be achieved only by introducing circumstance-based interventions. A public health approach that involves understanding community behavior, public communication, and community engagement is necessary to implement such interventions [5]. As the pandemic management strategies are individual and public-centered, it is crucial to understand the perception, concerns, and awareness of the population concerned toward the pandemic infections and situations [6]. At times of pandemics, investigating public opinion and viewpoints through traditional approaches like surveys and interviews comprise challenges in safety, reachability, and response rate [7].

Earlier researches affirm that during outbreaks social media was been widely utilized by the public to share their emotions and viewpoints towards the situation [8]. Analysis of social media content has been extensively performed to manage many health-related issues [9,10] and disease outbreaks [11]. Research confirms that in India, Twitter has facilitated social and political communication amid the COVID-19 pandemic [12]. Research in India also shows that social media use was positively correlated with people's inclination to take voluntary protective actions, and social media has a major impact on the public's and healthcare professionals' adoption of COVID-19 prevention strategies [13]. Social media information was also been researched during the COVID-19 pandemic in various other aspects. Earlier social media studies related to COVID-19 included the identification and prediction of cases, investigation of the information, public attitudes and emotions, psychological surveillance, and examining the validity of information in social media [14].

Although previous studies investigated the public discussions in social towards COVID-19, analysis of information in social media regarding COVID-19 third wave is not been published to the best of our knowledge. Previous research has confirmed that there will be a change in social media discussions and emotional expression towards any topic or event corresponding to the changes in reality [15]. Especially during the COVID-19 pandemic, since the situation was very uncertain and unpredictable, the attitudes and emotions of the public also changed accordingly based on various factors [16]. However, no studies were directed to examine Indian citizens’ perspectives on the COVID-19 third wave. Hence this study emphasizes analyzing the general public's opinion on social media content regarding the COVID-19 third wave and attempts to answer the following research questions (RQ) to understand the perception of the population.

RQ 1: What are the emotions shared by Indian citizens towards COVID-19 third wave?

RQ 2: What are the themes of discussion by Indian residents towards COVID-19 third wave?

Understanding public opinion on the pandemic situation will help emergency managers plan health promotion strategies and disseminate the right health information. A similar methodology using social media data can be utilized in the future for effective management of pandemic situations.

## Materials and methods

Data acquisition and pre-processing

**Figure 1:**
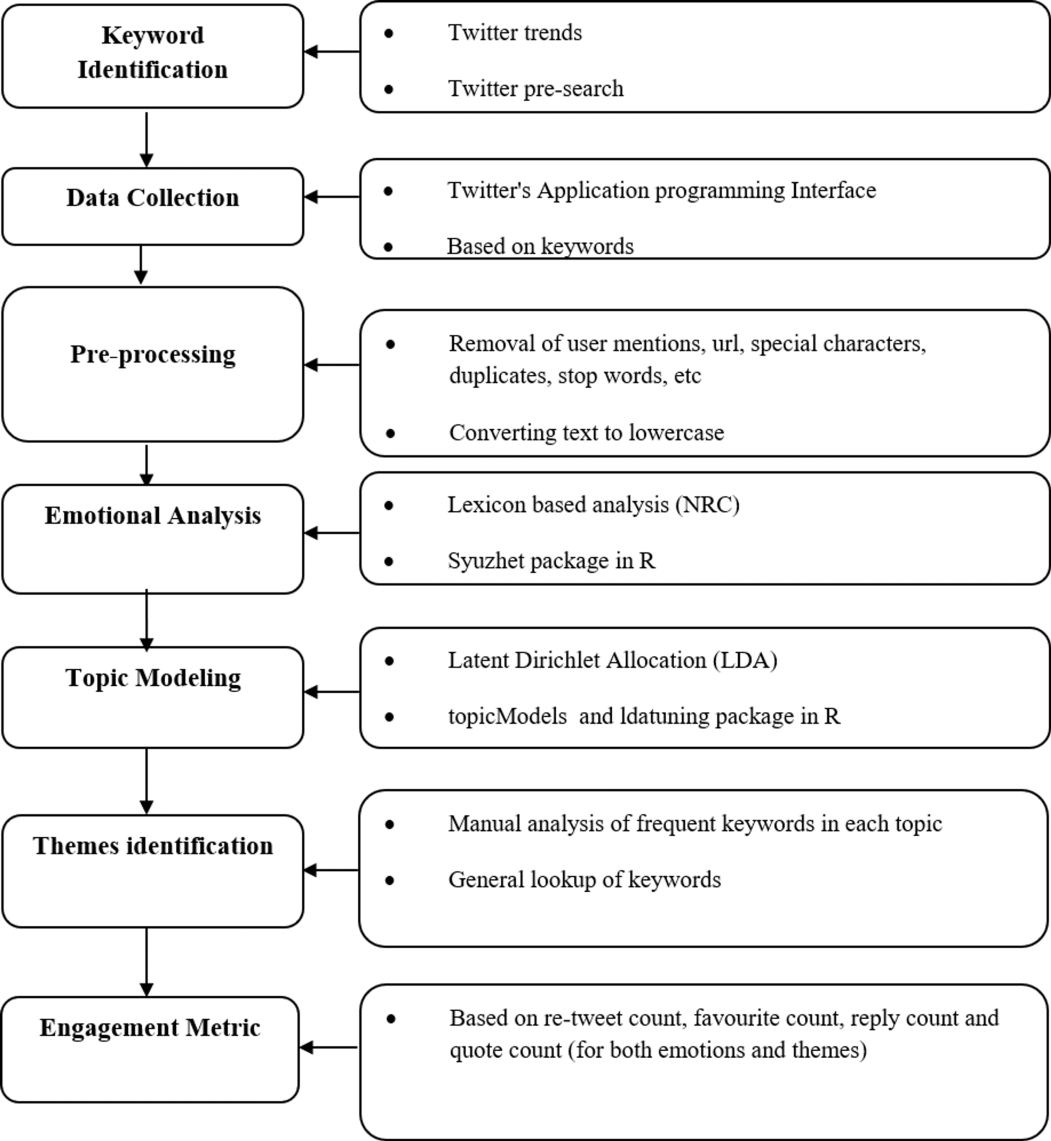
Methodological Framework

In this study, tweets were used as a major data source to analyze the viewpoints of Indian citizens towards COVID-19 third wave. Figure 1 depicts the methodological framework.

By using the “rtweet” package, the tweets were collected via Twitter Application Programming Interface (API) from June 1, 2021 to July 10, 2021. These dates were chosen for analysis since it was predicted that the third wave of COVID-19 infections might hit anytime during that period and there was an anticipation among people towards it. Twitter trends were used to identify the appropriate keywords related to the COVID-19 third wave. The keywords “thirdwave”, “deltaplusvariant”, “deltaplus”, “deltavariant”, “COVIDthirdwave”, “thirdwaveCOVID”, “COVID19thirdwave”, “3rdwave”, “COVID3rdwave”, “third_wave”, “newvariant”, “third wave”, “3rd wave” and “India” were used to extract the tweets. In text mining, preprocessing social media data is essential because it standardizes data and eliminates noise (such as emoticons, and misspellings), which enables models to receive more clean and structured input and increases analytical accuracy [17]. Pre-processing provides improved efficacy in topic modeling, and sentiment analysis [18]. The collected tweets were pre-processed by removing user mentions, external links, special characters, duplicates, and stop words, and also the text was converted to lowercase.

Emotion analysis

Emotion analysis was used to classify the text into various emotions and it gives emotion scores to every message. Eight different emotions, namely, joy, surprise, anticipation, trust, fear, anger, sadness, and disgust, were considered. These emotions are a part of Plutchik's wheel of emotions. Using the National Research Council (NRC) Lexicon, the tweets were categorized into emotions using the "Syuzhet" package in R (R Foundation for Statistical Computing, Vienna, Austria). The NRC Lexicon combines seven different lexicons to create a word-emotion association dictionary. Earlier research on Twitter emotional classification across several domains frequently employed the NRC Lexicon. NRC lexicon assigns every tweet an emotion score for each of the eight emotions.

Topic modelling

Topic modeling is a probabilistic approach that helps to sort out unorganized text into systematic groups [19]. Topic modeling, a computational technique, was used to determine the COVID-19-related themes or topics of discussion among the population. Among many topic models available, Latent Dirichlet Allocation (LDA) is one of the widely accepted, reliable, and standard techniques in many different domains and research areas [20]. The LDA technique has also been applied to social media data to aggregate and organize public conversations on a variety of health-related subjects [21]. TopicModels [22] and the ldatuning package in R were utilized to identify optimum topics and also to segregate tweets into different themes. Based on frequent words in each theme by the model and general lookup of random tweets from each category, the themes were categorized and aggregated manually and given appropriate names.

Engagement metrics

Engagement metrics analysis was done with respect to emotions and themes considering re-tweet count, favorite count, reply count, and quote count. These numbers show how available and engaging the tweet's content is with the general public. For instance, a tweet's greater favorite and retweet counts indicate that the information is more accessible to a wider audience. The more people respond to and cite a tweet, the more engaged the public is with that specific tweet. The reachability and engagement of the public towards the tweet are accessed using the favorite ratio, retweet ratio, quote ratio, and reply ratio. These reachability and engagement metrics in the context of emotions and themes were calculated using equations 1, 2, 3, and 4, respectively [23,24].

Favorite ratio = (Sum of favorite count pertaining to particular emotion or theme)/(Total number of tweets corresponding to that theme or emotion) Equation (1)

Retweet ratio = (Sum of retweet count pertaining to particular emotion or theme)/(Total number of tweets corresponding to that theme or emotion) Equation (2)

Quote ratio = (Sum of quote count pertaining to particular emotion or theme)/(Total number of tweets corresponding to that theme or emotion) Equation (3)

Reply ratio = (Sum of reply count pertaining to particular emotion or theme)/(Total number of tweets corresponding to that theme or emotion) Equation (4)

The higher the ratio, the higher the reachability and engagement of the public towards that tweet content/emotion.

## Results

A total of 11,849 tweets were extracted from the Twitter API using R programming from June 1, 2021 to July 10, 2021, inclusive. After eradicating unwanted tweets, texts, symbols, and duplicates, a total of 8933 tweets were considered for analysis.

Emotion analysis of COVID-19 third wave

The tweets were assigned scores for the eight emotions - anger, anticipation, disgust, fear, joy, sadness, surprise, and trust - based on the NRC Lexicon. It was found from the analysis that the citizens expressed multiple emotions in a single tweet. A maximum of 4180 tweets expressed anticipation, followed by 4070 tweets expressing fear and 4001 tweets expressing trust. Anticipation was expressed maximum may be because of the predictions and forecasting of COVID-19 third wave during that period. Fear was expressed due to the past bitter experiences undergone by the people during COVID-19's first and second waves. Table 1 shows the total number of tweets for each emotion, two example tweets for every emotion, and the most frequent words in tweets of each emotion. Two example tweets were selected at random from each emotion and this also gives an understanding of different contexts of emotional expressions.

**Table 1:** Number of tweets and example tweets in each emotions

S. No.	Emotions	Number of Tweets	Frequent Words	Example Tweet 1	Example Tweet 2
1.	Anger	3109	Hit, Inevitable, August, AIIMS, Threat	Anti-national elements of our country r spreading rumours regarding third wave of Corona. It is very unfortunate. As our country has very successfully combatted two waves as compared to advanced countries of d world, incase if by bad luck, India has d potential to tackle.	The Third Wave of COVID-19 can hit India in 6-8 weeks. The Country is still in shock from the horror of the Second wave. Even then only 3.6% of the Population is fully vaccinated Why does the Govt wants the people to suffer any further?
2.	Anticipation	4180	Start, Expected, Ready, Prepare, time	With our available resources and the ongoing generosity of donors, we supported our partner hospitals in #India. These donations will continue to support patients and ensure that hospitals are better prepared for any third wave of the #pandemic.	Better India should plan how to deal with the third wave of COVID in case if it comes.
3.	Disgust	1229	Deadly, Blame, Govt, Second, Bad	India is already suffering because of all the damage the second wave of COVID did & is doing, not even 3% of our population is vaccinated (& only adults have), there is another epidemic of black fungus going on, the third wave will hit soon & if another outbreak happens....	The highly-contagious #DeltaVariant of the novel coronavirus, which drove India's dreadful second wave of the pandemic, has further mutated, giving birth to the AY.1 or Delta Plus variant. So, does it lead to an even severe disease of #COVID19 ?
4.	Fear	4070	Government, Variant, Delta, Prevent, Risk	What's I am seeing after being in top five in coronavirus cases worldwide and so many deaths and suffering the Indians don't learn anything at all!. I am afraid what would the third wave will do in India	The speed at which people here in India went from being scared to be near each other, to let me take my mask off to talk to you right in your face is terrifying and why the #ThirdWave will come sooner than we realize. #COVID19 #DeltaVariant
5.	Joy	1642	Save, Safe, People, Please, Important	Happy to see a good VaccinationDrive on the first day of government s Free Vaccine Policy It will strengthen India s Fight against COVID Hope it will avoid any possibilities of a third wave We have already been through a lot	Good initiative Hope we don t see the third wave GOD BLESS TAMIL NADU AND INDIA
6.	Sadness	2618	Pandemic, Exam, Cancel, Repeater, Backlog	So depressing ! AIIMS Chief says Covid third wave inevitable, could hit India in 6-8 weeks	Sad to see India's daily #COVIDVaccination slumping to around 4 million jabs and less after an initial spurt which saw over 8 million.
7.	Surprise	1591	Surge, Tackle, Deal, Hope, Good	I am surprised a AIIMS student requesting for postponement of NEET when AIIMS cheif have already said that we can expect third wave from october	Many in India still refuse to wear masks even as experts warn of third COVID wave, finds survey. A new survey has found that 67% of respondents in India said there is low mask compliance in their areas.
8.	Trust	4001	Prepared, Director, Scientist, Children, Medical	Pledge to wear my mask to keep India safe and healthy and save us from the third wave!	If third wave of COVID catches up in India, we, only we the people of India would be responsible for it. Then, at that stage don't blame the Government, EC, religion etc. Just sufer it and embrace death willingly and happily. All the best fellow countrymen

On looking into some of the sample tweets from each emotion, the contexts of the emotional expressions were identified. People conveyed their anger towards rumors about the third wave and a lack of vaccination coverage; anticipation of generous support from co-citizens, and preparedness for the COVID-19 third wave; disgust towards the pandemic and black fungus infection; fear towards fungus attacks and warning from experts about the severity of the alarming third wave; joy towards free vaccine policy and the initiatives taken by the Government to prevent the third wave; sadness towards the lack of awareness regarding vaccination and the loss to the economy; surprise towards the postponement of examination and the irresponsible nature of some citizens regarding wearing masks; and trust towards the preparedness and medical facilities available in India.

Topic modelling

The tweets were analyzed using LDA topic models and it yielded eight topics. After examining these eight topics manually, they were grouped into the following six themes. 

Theme 1: Preventive measures for COVID-19 third wave - Twitter messages that had information on measures like wearing masks, hygiene, and social distancing that prevent the spread of COVID-19 at the individual and population levels. 

Theme 2: Information on the delta variant virus - Tweets containing details about the COVID-19 delta variant, how contagious it is, and how effective the vaccine is against it.

Theme 3: Information on various COVID-19 waves - Tweets that provided details and compared the impacts, public behavior, and policy changes during the first and second waves of COVID-19.

Theme 4: Knowledge about vaccination - Messages containing facts about the availability of vaccinations and their accessibility in India, their dosages and effectiveness, how to register on the CoWIN app, and immunization certificates.

Theme 5: Expert opinion - Twitter messages that had any information regarding the COVID-19 scenario shared by authorized institutions and experts of different fields such as epidemiologists, immunologists, and public health researchers.

Theme 6: Views on education - Conversations and opinions on the social and emotional effects of disrupted education systems due to the pandemic in the short and long term and also the advantages and disadvantages of e-learning.

Each tweet was assigned to a particular theme mentioned above. It was found that 34% of the tweets focused on the preventive measures for COVID-19 third wave (Theme 1), 19% of the tweets dealt with expert opinion (Theme 5), 15% of the tweets showed interest in information on the delta variant virus (Theme 2), 12% of the tweets emphasized on information on various COVID-19 waves (Theme 3), and 10% each of the tweets concentrated on facts about vaccination (Theme 4) and views on education (Theme 6). Table 2 displays the number of tweets in each theme, highly frequent words in each theme, and two example tweets that were selected at random corresponding to each theme.

**Table 2:** Number of tweets and example tweets in each theme

S.No	Themes	Number of tweets	Keywords	Example Tweet 1	Example tweet 2
1.	Theme 1	2997	People, vaccination, prepare, lockdown, think, mask, help, children, waves, fight, ready, oxygen, plan, third, hope, time	Emergency for third wave warning Delta Variant. very carefully for Children, Adult and Elderly seniors citizen stay mask and stay home !!	Most hospitals in India are not equipped with the technology to store oxygen in its liquid form and, therefore, use cylinders to store it in its gaseous form
2.	Theme 2	1316	Variant, delta, cases, wave, new, plus, delta variant, deltaplus variant, already, COVID, cause, come	COVID Studies indicate the challenges to come a possible ThirdWave infections among children deaths from fungal infections and the emergence of mutating highly transmissible virus variants	A worrying Delta sweep puts unvaccinated people at higher risk
3.	Theme 3	1107	Wave, COVID, third, second, first, news, report, severe, speak, September, mid, august, early, times, see	Third wave of COVID can hit India in August may reach peak in September says SBI report	Third COVID wave in India to be a 'ripple' if there's no fast-spreading mutant
4.	Theme 4	899	Vaccination, vaccine, vaccinated, population, prevent, must, day, can, even, yet, virus	India versus COVID, our vaccine strategy, the fear of a third wave, super-mutations, the need for boosters, the need to tweak vaccines, should we be worried by Delta Plus and how do we	Proud that India is producing and exporting vaccines Will focus on improving everyones health
5.	Theme 5	1698	Expert, pandemic, threat, today, brace, impact, health, hit, inevitable, appropriate, aiims, warns, director, chief, October, infection, restriction	As the second COVID wave leaves India devastated amid the third wave warning those who were severely hit by the deadly respiratory disease must also keep the health of their kidneys in check top health experts advised	COVID The secondwave of coronavirus infection might be showing signs of receding from parts of India but there are serious concerns that the ThirdWave might affect children
6.	Theme 6	860	Exam, student, year, offline, cancel, university, situation, please, want, conduct	There is threat of COVID third wave in India, then I don't understand why want to take exam of class 12 private student.They don't care abt our life whether we live or die. So I req. cbse to cancel private candidates exam	Dear Sir Now the third wave of corona virus is going on At the moment universities want to conduct exams offline The university is not thinking about the students

In Theme 1, people consciously spoke about preventive measures like wearing masks, vaccination, child safety, extension of lockdown, and government initiatives to safeguard children. Theme 2 conversed about the delta variant virus, risks that will be encountered due to COVID-19 third wave, and severity of both virus and fungal infection. In Theme 3, Indian residents shared their views on COVID-19's first, second, and third waves, the time at which the third wave will hit India, and the anticipated duration of the third wave. Theme 4 conferred about the past and present status of vaccination, the CoWIN app, covaxin, covishield, sputnik, and also concerns regarding the availability of vaccines. Theme 5 examined about All India Institute of Medical Sciences (AIIMS) and Indian Council of Medical Research (ICMR) reports, expert views on COVID-19 third wave, and warnings to people. Theme 6 talked about student life, examination cancellation, offline/online mode of exam, and the possibility of exams being conducted.

Engagement metrics

**Figure 2:**
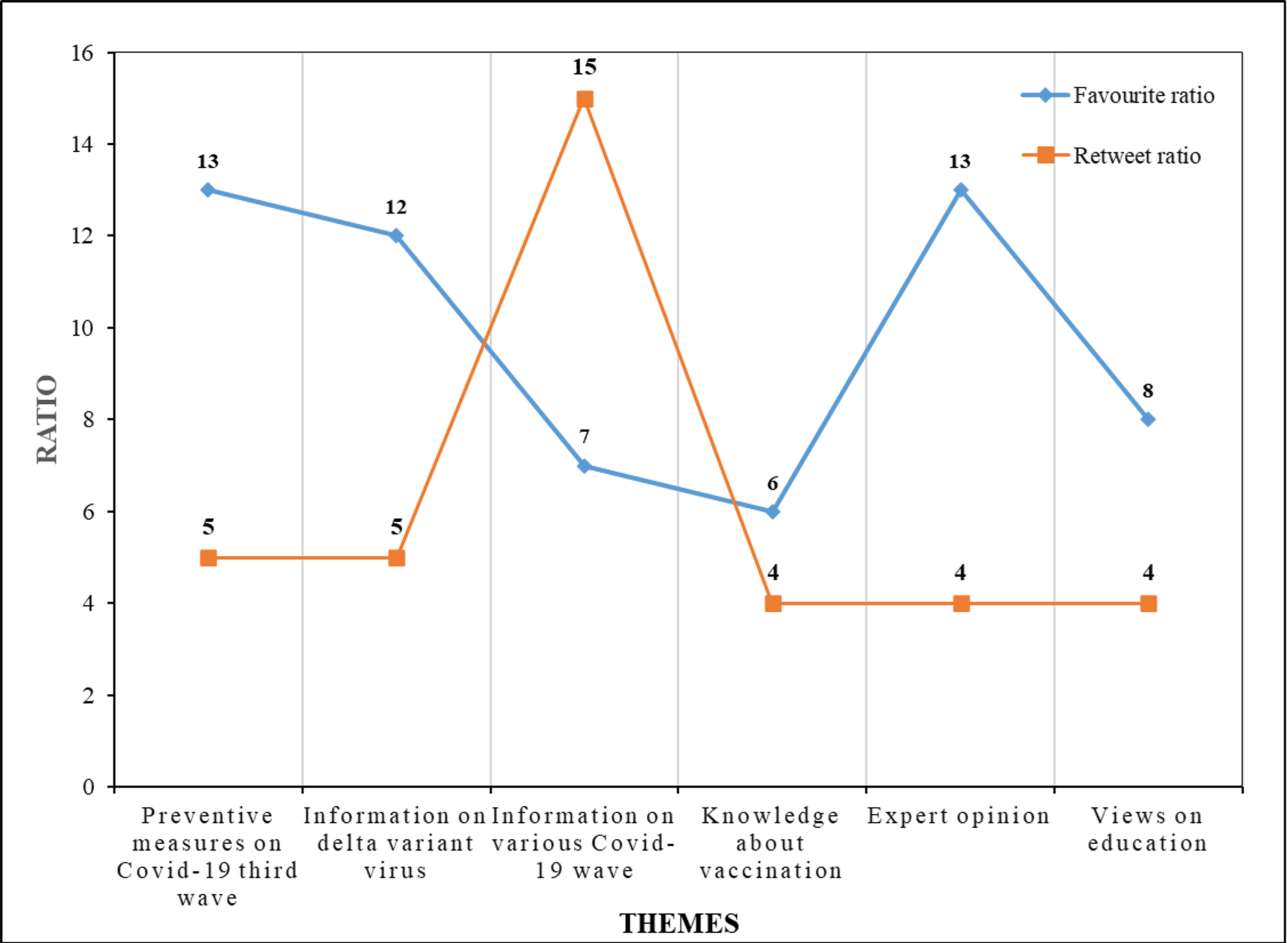
Theme-wise retweet and favourite ratio

**Figure 3:**
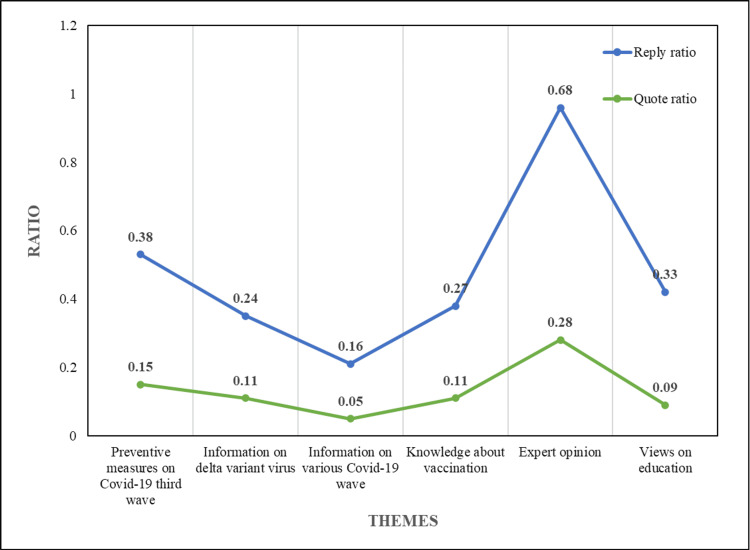
Theme-wise reply and quote ratios

The engagement metrics (favorite ratio, retweet ratio, quote ratio, and reply ratio) of various emotions and themes are shown in Table 3, Figure 2, and Figure 3

**Table 3:** Average engagement metrics of COVID-19 third wave emotions

Emotional polarity	Favorite ratio	Retweet ratio	Quote ratio	Reply ratio
Anger	14	7	0.19	0.40
Anticipation	9	3	0.15	0.43
Disgust	18	15	0.16	0.45
Fear	11	6	0.16	0.33
Joy	16	5	0.16	0.52
Sadness	12	9	0.14	0.37
Surprise	16	4	0.18	0.45
Trust	13	8	0.13	0.39

Table 3 makes it evident that tweets expressing disgust had the highest favorite ratio (18) and retweet ratio (15). Anger tweets had the maximum Quote ratio (0.19) and joy tweets had the maximum reply ratio (0.52). A favorite ratio of 18 means that each tweet with disgust is likely to receive 18 likes on average. Similarly, a retweet ratio of 15 indicates that, on average, every disgust tweet was likely to be retweeted 15 times. Higher favorite and retweet ratios mean that the tweets with the particular emotions have reached and have been accessed by a wider audience. Conversely, larger quote and reply ratios indicate that more individuals were actively participating in the conversation and actively responding to tweets expressing a certain emotion. Even though the anticipation emotion was expressed maximum in the tweets by the public, the disgust tweets scored the highest favorite and retweet ratio. It means that the disgust tweets reached and were accepted by the maximum number of people, maybe because people were annoyed due to the non-responsiveness of some citizens. Tweets with anger had a higher quote ratio due to the fact that people were spreading rumors and making the public panic. Tweets with joy had a higher reply ratio because people were contented with the measures taken by the Indian Government to face the COVID-19 third wave

From the analysis of Figure 2 and Figure 3, it is found that Theme 1 and Theme 5 had the maximum favorite ratio, maybe because people trusted and were eager to hear from experts on precautions and measures taken towards COVID-19 third wave. Theme 3 scored a higher retweet ratio as it had information regarding the past, present, and forthcoming COVID-19 waves, and maybe people wanted to share that information to create awareness. As Theme 5 discussed vaccination and related issues, it had the highest quote and reply ratios.

## Discussion

This study concentrated on the conversation themes and emotional expressions found in Twitter posts on the COVID-19 third wave between June 1, 2021, and July 10, 2021, prior to the outbreak of the third wave in India. This study made it easier to comprehend how Indians are talking and feeling about COVID-19 and how ready they are for the next wave on Twitter. Additionally, the public's favorited, shared, and discussed tweets were examined for their emotional content and themes.

During the study period, anticipation was expressed in the maximum number of tweets followed by fear and trust emotions. Maximum levels of trust, fear, and anticipation were also reported in earlier research that examined emotions in several COVID-19 scenarios [25,26]. During COVID-19 scenarios, India has been experiencing a surge of misinformation and fake news in social media [27]. But later, through multilingual infographics, official accounts like @MoHFW_INDIA, and campaigns like #IndiaFightsCorona, the Indian government used Twitter to counteract misinformation and ensure widespread reach while promoting urgent COVID-19 preventive steps. Experts say that increasing social media messaging from reputable and authoritative sources is one of the best ways to prevent false information on social media [28] and the government followed the same to combat misinformation. Social media misinformation and the detrimental effects of previous COVID-19 waves could be the cause of the public's fear. Governments initiative towards vaccines and information dissemination might be reason for anticipation and trust emotional expression.

According to the results of topic modeling, the maximum number of tweets that were posted during the considered period was on preventive measures that need to be followed to control COVID-19 infections. This may be because, public health organizations and the government utilized social media as a tool to disseminate information about COVID-19-related events, risks, and preventive measures that may be taken to slow the spread of the disease globally [29] and also in India [30]. The studies that analyzed the content of Twitter messages posted in India during COVID-19 scenarios also confirmed that there was a maximum number of tweets related to preventive measures [31,32]. Regarding the reachability of messages with respect to emotions, tweets with disgust expressed were maximum liked and re-tweeted. The messages that had anger emotions had the maximum number of comments, which means they were widely discussed.

False information about COVID-19 was also quickly circulating on social media during the pandemic. Studies also confirm that Twitter handles including those of organizations and celebrities also participate in the creation of new tweets and the retweeting of misinformation [33]. Similar to several other nations, disinformation gains fresh impetus in India also, with the growing usage of social media playing a significant role [34]. In India, during the pandemic, extensive fear, false information, and chaos that are fueled by intense usage of social media, print, and broadcast media coverage were also reported [35]. This fear and misinformation may also be the reason for more discussion and engagement with disgust and anger messages. The public's acceptance of the expert opinions and messaging about COVID-19 during the consideration phase may have stemmed from their trust in the experts' judgment and their anticipation of the actions taken to stop the virus from spreading.

Our study's limitation is that the findings may not accurately reflect the views of the entire Indian community, as data was collected from Twitter. India's population is not fully represented on Twitter because the majority of its users are educated and are those who are connected to the internet. While there is a greater chance of temporal fluctuations in emotions and themes during epidemics, this study examined the emotions and conversation topics of populations at a single moment in time. Another limitation is that since the approach in this study is computational and unsupervised, it only provides a fundamental, basic insight into public opinion. In the future, utilizing mixed method analysis (a combination of quantitative and qualitative) and creating training sets tailored to specific locations and outbreak contexts for emotional classification will provide a complete understanding of the public's perspective.

The study's findings provide essential insights into people's beliefs and expectations. Government and public health organizations can design health information dissemination strategies based on people's needs. A similar methodology can be used in any emergency scenario to understand the population’s needs and perceptions, based on which health communication and health promotion initiatives can be strategized.

## Conclusions

This study used the Twitter platform to collect social media posts as data. Text mining and machine learning techniques were adopted to segregate tweets into various emotions and themes. The results reveal that anticipation was a predominant emotion expressed by people. The results confirm that during outbreaks people from India used social media to share their emotions and also seek information. Since social media is used by more Indian residents to share their views, the government and healthcare stakeholders can also use the same to disseminate the right information. Health information communication on Twitter can be planned so that scientific information takes the place of disparate viewpoints that would otherwise dominate social media content. When people receive accurate and validated information on virus prevention and transmission at the appropriate moment, it will enhance their behavioral response and lessen fear. Further, considering the viewpoints expressed in social media, the government and health professionals can take necessary actions based on the population’s need to handle the pandemic situation in an efficient manner. In the future, social media can be explored using similar methods, and the information can be used for planning effective communication with the public, promoting health education, and responding to public health emergencies.
